# Shared microbiological and immunological patterns in periodontitis and IBD: A scoping review

**DOI:** 10.1111/odi.13843

**Published:** 2021-03-23

**Authors:** Giacomo Baima, Alessandro Massano, Erminia Squillace, Gian Paolo Caviglia, Nurcan Buduneli, Davide G. Ribaldone, Mario Aimetti

**Affiliations:** ^1^ Department of Surgical Sciences C.I.R. Dental School University of Turin Turin Italy; ^2^ Department of Medical Sciences University of Turin Turin Italy; ^3^ Department of Periodontology School of Dentistry Ege University İzmir Turkey

**Keywords:** dysbiosis, gut, immunity, microbiome, periodontal diseases

## Abstract

**Objectives:**

To extract the microbiological and immunological evidence underpinning the association between periodontitis and inflammatory bowel disease (IBD).

**Methods:**

Relevant articles were sorted through a systematic search on PubMed, Embase, Scopus and Web of Science up to October 2020. Available evidence was grouped in three different clusters: (a) studies that examined oral microbial alterations in IBD patients; (b) studies that investigated intestinal dysbiosis in patients with periodontitis; and (c) evidence for a shared immunological pattern between the two conditions.

**Results:**

A total of 15 studies involving 1,171 patients were included. Oral microbiome, either subgingival or salivary, was consistently altered in patients with IBD compared to healthy subjects (a) Additionally, gut dysbiotic microbiota of IBD patients was colonized by pathobionts from oral origin, either via haematogenous or enteric route. Suffering from periodontitis is associated with lower alpha diversity in the gut microbiome (b) Lastly, both IBD and periodontitis are characterized by similar expression patterns of inflammatory cytokines at the gingival and gut levels that are exacerbated when both diseases are present (c).

**Conclusions:**

Periodontitis and IBD share common dysbiotic and immunological traits. Well‐designed preclinical models and longitudinal cohort studies are required to better explore the causal pathways between the two conditions (PROSPERO CRD42020194379).

## 1 INTRODUCTION

Inflammatory bowel disease (IBD) is a group of chronic idiopathic disorders, including two major entities: Crohn's disease (CD) and ulcerative colitis (UC) (Actis et al., 2019). Both are characterized by chronic inflammation of the gastrointestinal tract and by periods of activity, quiescence, and relapse (Kaser et al., 2010). It is estimated that nearly 6.9 million people are living with IBD worldwide, mostly in Europe (2.2 million) and North America (1.5 million), with the number of incident cases on the rise (GBD, 2017). The clinical aspect is represented by abdominal pain, diarrhoea, rectal bleeding, anaemia and weight loss including extraintestinal manifestations (Gomollòn et al., 2017). Knowledge about the aetiopathogenesis of IBD is still limited, but it is supposed that its onset originates from genetic and environmental factors, together with impaired intestinal permeability, gut dysbiosis and immunological dysregulation (de Souza & Fiocchi, 2016).

Periodontitis is a biofilm‐induced chronic inflammatory disease of tooth supporting structures that leads to progressive loss of attachment and alveolar bone destruction (Tonetti et al., 2018). Periodontitis is the major cause of tooth loss in adults, with an estimated prevalence of 30%–50% of the population (Frencken et al., 2017). At the initial stages, clinical signs and symptoms can be lacking or very mild—such as gingival inflammation and bleeding. When periodontal tissue destruction proceeds, the disease results in tooth mobility, drifting, flaring and finally loss of the affected tooth (Könönen et al., 2019). Regarding its pathogenesis, it is currently considered that microbial aetiologic factors induce a series of host responses that mediate inflammatory events in genetically susceptible patients, with environmental risk factors playing a modifying role (Van Dyke et al., 2020). Periodontitis has been linked with various systemic diseases/conditions and their negative consequences, mainly through bacteraemia and low‐grade chronic inflammation (Romandini et al., 2021).

Recent studies have provided significant evidence for the association between inflammatory bowel disease and periodontitis (Papageorgiou et al., 2017). However, the underlying connection mechanisms remain unclear. Hypotheses mainly focus on a common bacterial aetiology and a connection between the immune pathways involved in both diseases. In fact, dysbiosis occurs in periodontitis such as in IBD, and oral microbiome is likely to move to the gut and affect the gastrointestinal microbiome (Kitamoto et al., 2020; Lira‐Junior & Figueredo, 2016). Moreover, both diseases are characterized by an excessive and non‐self‐limiting immune response towards the dysbiotic microbiome (Kaser et al., 2010).

Although meta‐epidemiological evidence is now available (She et al., 2020), the study of the biological association between the two diseases still presents remarkable heterogeneity in methods and findings (Agossa et al., 2017). To explore such a composite body of knowledge, scoping reviews may be preferred over systematic reviews because of the broadness of their coverage and their ability of mapping the evidence to discuss advancements and limitations (Munn et al., 2018). Therefore, the present scoping review was conducted to investigate the relationship between periodontitis and IBD at both immunological and microbiological levels, and to identify gaps in the available evidence to be implemented by future research.

## 2 METHODS

### 2.1 Study design and reporting

The present scoping review was structured according to the PRISMA (Preferred Reporting Items for Systematic Reviews and Meta‐Analyses) Extension for Scoping Reviews checklist. A detailed protocol has been registered on PROSPERO before the commencement of the work (CRD42020194379).

### 2.2 Search strategy

The following electronic databases were searched in duplicate by 2 review authors (GB and AM): MEDLINE (via PubMed), Embase, Scopus and Cochrane Library. No restrictions on language or date of publication were employed when searching the electronic databases. General controlled vocabulary (MeSH terms) and keywords were chosen, including “bacteria”, “microbiome”, “microbiota”, “dysbiosis”, “inflammatory bowel”, “ulcerative colitis”, “Crohn's disease”, “periodontal disease”, “periodontitis” and “cytokines”. In addition, two review authors double‐checked the bibliographies of all included studies and previous reviews.

### 2.3 Eligibility criteria and selection of the manuscripts

The primary scope of this review was sorting studies presenting microbiological and immunological analysis in patients with both periodontitis and IBD. Due to the paucity of entries retrieved, studies investigating oral microbial/immunological changes in IBD patients and gut microbial/immunological variations in periodontitis patients were also included. Thus, only original comparative studies of human populations, case–control studies, cohort studies or intervention studies with the concomitant analysis of clinical and immunological or microbiological parameters at oral and gut levels were selected for this review. Case reports, literature reviews, editorials, animal studies and in vitro experiments were excluded, and so were studies dealing with probiotic supplements.

Titles and abstracts of all reports identified through the searches were screened independently by the two review authors. For studies appearing to fulfil the inclusion criteria, the complete manuscript was obtained. The full texts obtained from various electronic sources and manual searching were thereby analysed independently for eligibility check. The reasons for exclusion of studies were recorded.

### 2.4 Data extraction and synthesis

A two‐step procedure was used to extract data from the included studies independently by two reviewers. Initially, descriptive data were tabulated with respect to population, exposures, controls and outcomes. Second, each selected study was re‐analysed and main findings were critically reviewed in the context of the available literature. The studies were then grouped in three different sections with respect to the aim and the initial population recruited. A first pool of articles set the spotlight on the analysis of oral microbiome in patients suffering from IBD. A second group focused on the intestinal microbial alteration that can be encountered among periodontal patients. Due to paucity of evidence, we included the studies in which gut microbiome in IBD patients was compared to oral flora regardless the presence of periodontitis. A third cluster was aimed at gathering the evidence about common immunopathological features between the two diseases.

## 3 RESULTS

The literature search resulted in 688 manuscripts, and after the removal of duplicates and those that did not meet the inclusion criteria, 28 were considered for full‐text review. After full‐text reading, 13 articles were excluded (Table S1) and Figure S1 presents a PRISMA flow diagram outlining the study selection process. The search method finally yielded 15 articles relevant to the research questions. Analysis of the geographic distribution revealed that 5 studies were carried out in Brazil, 4 in the United States and 2 in Germany, while the others originated from Sweden, China and Japan.

### 3.1 Oral microbial alterations in IBD patients

Studies investigating the impact of IBD on oral or periodontal microbiota are summarized in Table 1. Nine studies were selected, with a sample size ranging from 40 to 147.

**TABLE 1:** General overview of studies investigating oral microbiome alterations in IBD patients

Author	Study design	Aims	Subjects	Periodontitis definition	Periodontal assessment		Tissue sampled		Laboratory analysis		Key findings	
Xun et al., 2018	Case–control study	To investigate oral dysbiosis in IBD	57 UC, 13 CD, 25 HC	NA	NA		Saliva		16S rRNA gene sequencing (Illumina)		Enrichment of Streptococcaceae and Enterobacteriaceae in UC; Veillonellaceae in CD; *Streptococcus* and *Veilonella* in both groups, when compared to HC Depletion of Lachnospiraceae, Porphyromonadaceae and *Prevotella* in UC; Porphyromonadaceae, Neisseriaceae, *Neisseria* and *Haemophilus* in CD; *Porphyromonas* in both groups, when compared to HC	
Schmidt et al., 2018	Cross‐sectional	To investigate periodontal pathogenic bacteria in IBD	29 UC, 30 CD, 59 HC	All patients with moderate or severe PD	PPD, CAL, PBS		Subgingival plaque and GCF		Semiquantitative PCR		Significantly lower prevalence of *E*. *nodatum* and *E*. *corrodens* in IBD compared to HC Higher prevalence of *E*. *corrodens* in CD compared to UC	
Kelsen et al., 2015	Comparative study	To investigate subgingival microbiota in paediatric CD patients initiating therapy and after 8 weeks	Discovery cohort: 35 CD, 46 HC. Validation cohort: 44 CD, 31 HC	NA	NA		Subgingival plaque		16S rRNA gene sequencing		Enrichment of *Capnocytophaga*, *Rothia*, and *TM7* in CD at baseline. 8 weeks of therapy were sufficient to return these taxa to healthy levels *Alloprevotella*, *Fusobacterium*, *Porphyromonas* and *Prevotella* decreased in the antibiotic‐exposed CD group, compared to CD subjects not using antibiotics, at baseline and after 8 weeks of therapy	
Said et al., 2014	Cross‐sectional	To analyse the salivary microbiome alterations in IBD patients	21 CD, 14 UC, 24 HC	NA	NA		Saliva		454 pyrosequencing of the bacterial 16S rRNA gene		Higher Bacteroidetes*, Prevotella* and *Veillonella* in CD and UC groups as compared to HC Lower Proteobacteria*, Streptococcus* and *Haemophilus* in CD and UC groups as compared to HC Lower *Neisseria* and *Gemella* in CD group as compared to HC	
Brito et al., 2013	Cross‐sectional	To analyse the subgingival microflora composition of IBD patients with untreated CP	15 CD and CP, 15 UC and CP, 15 CP	At least 5 inflamed sites with PPD ≥ 5 mm and CAL ≥ 3 mm in different teeth	PPD, CAL, presence of plaque, BoP		Subgingival plaque		Checkerboard DNA– DNA hybridization technique		1. Gingivitis sites: Higher *P. melaninogenica*, *S, anginosus*, *S, mitis*, *S. mutans* and *T, denticola* in CD compared to UC. Higher *P. melaninogenica*, *S. anginosus* and *S. mutans* in CD compared to controls. Lower *S. anginosus* and *S. mitis* in UC compared to controls 2. PD site: Higher *B, ureolyticus*, *C. gracilis*, *P. melaninogenica*, *S. anginosus*, *S. intermedius*, *S. mitis* and *S. mutans* in CD compared to UC; also higher in CD compared to controls (except for *S. mitis*)	
Docktor et al., 2012	Case–control study	To study oral microbiome of children and young adults with IBD	40 CD, 31 UC, 43 HC	NA		NA		Tongue and buccal mucosal brushings		HOMIM: 16S rRNA‐based oligonucleotide reverse capture microarray		Decreased overall diversity of tongue samples in CD cohort when compared to controls Loss of probe activity of Fusobacteria and Firmicutes in tongue samples of CD; loss of probe signal of Fusobacteria in UC; rise of Spirochaetes, Synergistetes, and Bacteroidetes in UC, when compared to HC
Stein et al., 2010	Cross‐sectional	To examine periodontal pathogens in CD patients' pockets	147 CD	NA		Missing teeth, PI, GI, mean PPD, mean CAL, BoP, and CPITN values		Subgingival plaque		Dot‐blot hybridization		*A. actinomycetemcomitans* in 76.9% of CD, *P. gingivalis* in 62.6%, *P*. *intermedia* in 79.6% and *T*. *forsythia* in 64.6. *C. rectus* had the highest frequency, being found in 94.6% of the patients. Age, GI, PI, smoking and all bacteria to be potentially relevant risk factors for PD and CAL in univariable analyses
Meurman et al., 1994	Cross‐sectional	To investigate *Lactobacilli* and Yeast counts in patients with active CD and in remission	32 active CD, 21 CD in remission	NA		PI, GI, vertical bone pocket, decayed and filled surfaces of all teeth		Saliva		Oricult‐*N* and Dentocult‐LB dip slides were inoculated to cultivate salivary yeasts and lactobacilli and *S. mutans* counts		Higher salivary yeast counts among patients with active CD Higher counts of salivary *Lactobacilli* and *S. mutans* counts among patients with active CD
Van Dyke et al., 1986	Cross‐sectional	To investigate the role of infectious agents from periodontal lesions in IBD patients	10 IBD with PD, 10 IBD with no PD, 10 adult PD, 10 HC	NA		Gingival erythema, SoP, BoP, bone loss		Subgingival plaque		Dark‐field microscopy		The predominant isolate from IBD patients was a Gram‐negative rod (genus *Wolinella*) In periodontal disease‐free IBD patients, the bacterial load in the gingival sulcus was greatly reduced from that of the IBD group with PD Serum‐mediated inhibition of neutrophil chemotaxis in CD and UC

Abbreviations: BoP: bleeding on probing; CAL: clinical attachment loss; CD: Crohn's disease; CP: chronic periodontitis; CPITN: community periodontal index of treatment needs; GCF: gingival crevicular fluid; GI: gingival index; HC: healthy controls; IBD: inflammatory bowel diseases; PBS: papilla bleeding score; PCR: polymerase chain reaction; PD: periodontitis; PI: plaque index; PPD: probing pocket depth; SoP: suppuration; UC: ulcerative colitis; VPI: visible plaque index.

Xun et al., (2018) assessed dysbiosis of the oral microbiome in saliva samples of 57 UC patients, 13 patients with either active or remissive CD, and 25 healthy adults. 16S rRNA sequencing (Illumina) revealed a distinct metagenomic signature of both UC and CD in saliva. The investigation detected a distinctive enrichment of Streptococcaceae and Enterobacteriaceae in UC and Veillonellaceae in CD, accompanied by depletion of Lachnospiraceae and Porphyromonadaceae in UC and Neisseriaceae in CD. At genus level, *Prevotella* was depleted in UC, and so were *Neisseria* and *Haemophilus* in CD. Additionally, there was an enrichment of *Streptococcus* and *Veillonella* and a depletion of *Porphyromonas* in both UC and CD. Alpha diversity was significantly lower in both UC and CD. Interestingly, most of these taxonomic variation tendencies were encountered in the gut of IBD patients.

Saliva was also sampled by Said et al., (2014), in a cohort of 21 CD, 14 UC patients, and 24 healthy controls (HCs). Phylum *Bacteroidetes* was significantly higher in both of the CD and UC groups compared with the HC; the same trend was observed for genera *Prevotella* and *Veillonella*, while Proteobacteria and *Streptococcus* and *Haemophilus* followed the opposite direction. Two other genera, *Neisseria* and *Gemella*, were also found to be significantly lower in the CD group than the HC. The same approach was adopted in an early study by Meurman et al., (1994), which employed bacterial cultures to identify salivary microbiological features in CD patients with active or inactive disease. It was found that salivary yeast, *Lactobacilli* and *Streptococcus mutans* counts were higher in patients with active than those with inactive disease (Menegat et al., 2016).

Another subset of articles examined microbial alterations in subgingival plaque. In the study of Schmidt et al., (2018), subgingival flora was pooled from 59 IBD patients and 59 HCs. Semiquantitative polymerase chain reaction (PCR) for bacteria detection demonstrated significantly lower prevalence of *Eubacterium nodatum* and *Eikenella corrodens* in IBD compared to HC. Moreover, the prevalence of *E*. *corrodens* was significantly higher in CD compared to the UC group. Brito et al., (2013) conducted a similar experiment on 45 patients presenting both untreated periodontitis and CD, untreated periodontitis and UC, and patients with untreated periodontitis only. Checkerboard DNA–DNA hybridization showed that periodontitis sites harboured significantly higher concentrations of *Bacteroides ureolyticus*, *Campylobacter gracilis*, *Prevotella melaninogenica*, *Staphylococcus aureus*, *Streptococcus anginosus*, *Streptococcus intermedius*, and *Streptococcus mutans* in CD than UC and controls. Small differences could be detected also in gingivitis sites. On the other hand, Stein et al., (2010) examined periodontal pathogens in subgingival plaque of CD patients stratified for CARD15‐gene mutations. Among all 147 patients, the frequency of detection was 76.9% for *Aggregatibacter actinomycetemcomitans*, 62.6% for *Porphyromonas gingivalis*, 79.6% for *Prevotella intermedia* and 64.6% for *Tannerella forsythia*. *Campylobacter rectus* had the highest frequency, being found in 94.6% of the patients. No control group was assessed.

The cross‐sectional investigation by Kelsen et al., (2015) characterized the subgingival microbiota in paediatric patients with active or non‐active CD. 16S rRNA sequencing identified 17 genera as candidate biomarkers: *Capnocytophaga*, *Rothia* and *TM7* were more abundant in CD patients than the healthy controls. It was also found that both antibiotic exposure and disease state were associated with differences in bacterial community composition. Likewise, Docktor et al., (2012) studied the oral microbiome of children and young adults with IBD in comparison with HC, by sampling tongue and buccal mucosal brushings. Next‐generation sequencing highlighted that tongue samples in CD showed a significant loss of probe activity from the two phyla, Fusobacteria and Firmicutes. For UC patients, similar significant loss of probe signal was noted in Fusobacteria, whereas those of Spirochaetes, Synergistetes and Bacteroidetes were all increased as compared with the controls.

Lastly, Van Dyke et al., (1986) observed differences in oral microbiome in IBD patients compared to both periodontitis patients and HC. Using cultural method and dark‐field microscopy, the authors reported predominance of a Gram‐negative rod consistent with genus *Wolinella* from IBD patients. Interestingly, in the periodontal disease‐free IBD patients the bacterial load in the gingival sulcus was significantly less than that of the IBD group with periodontal disease.

### 3.2 Intestinal dysbiosis in periodontitis patients

**FIGURE 1:**
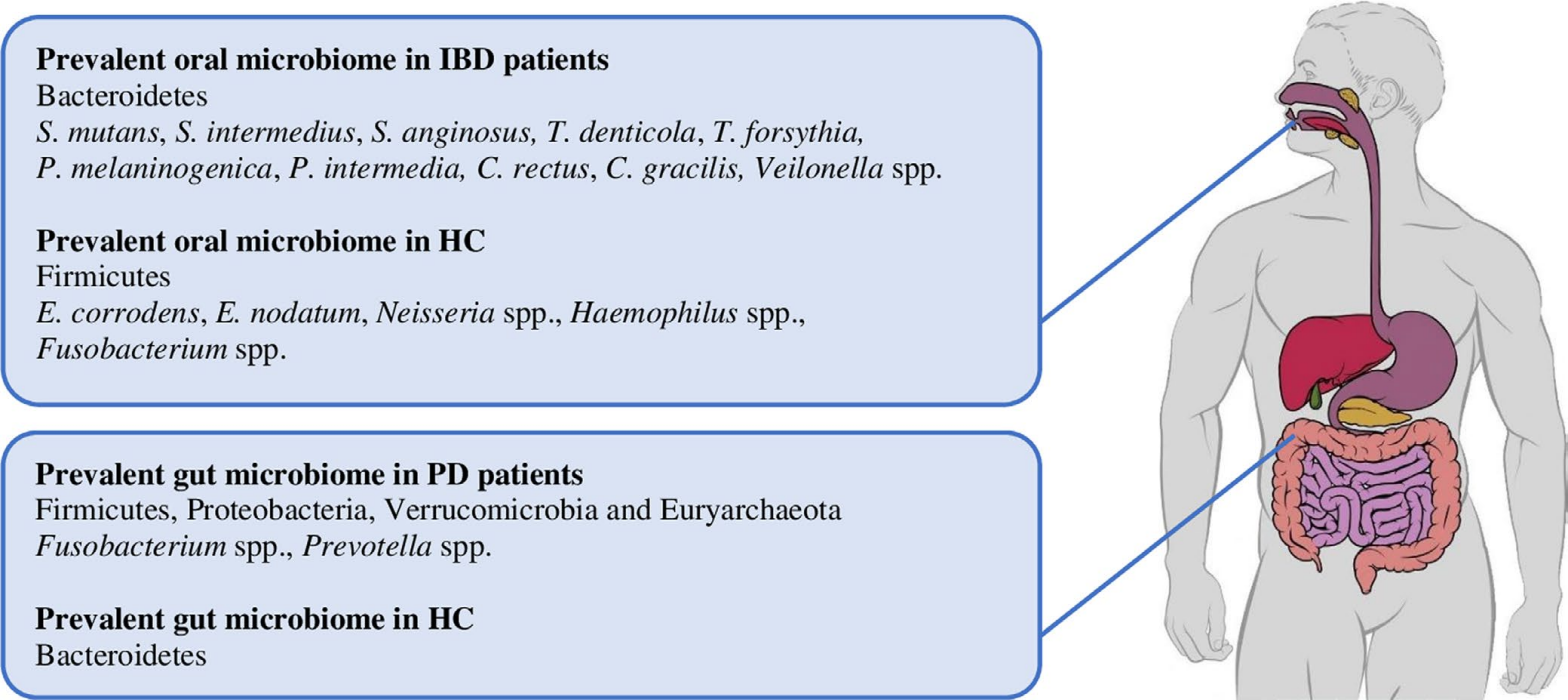
Prevalent oral and gut microbials in inflammatory bowel disease (IBD) and PD

Table 2 summarizes the evidence about intestinal microbial alterations in relation to oral‐derived bacteria, while Figure 1 depicts the prevalent bacterial clusters encountered in oral and gut environments in IBD and periodontitis. Only one study comparing the gut microbiome of individuals with different periodontal conditions was found.

**TABLE 2:** General overview of studies investigating gut dysbiosis in relation to oral bacteria

Author	Study design	Aims	Subjects	Periodontitis definition	Periodontal assessment	Tissue sampled	Laboratory analysis	Key findings
Lourenςo et al., 2018	Case–control study	To characterize the gut microbiome of individuals with different periodontal conditions	7 PH, 14 G, 23 CP	PD: > 10% of teeth with PD and CAL ≥ 5 mm with BOP G: > 10% of sites with BOP and/or GI, no PD or CAL > 3 mm, although PD	Full‐mouth protocol: PPD, CAL, BoP, GI	Stool samples	Illumina MiSeq Sequencing	Lower alpha diversity in the gut microbiome of individuals with CP Firmicutes, Proteobacteria, Verrucomicrobia and Euryarchaeota increased, whereas Bacteroidetes decreased in patients with PD compared to PH *Prevotella*, *Comamonadaceae* and *Lactobacillales* detected in higher numbers in G, while Bacteroidales predominant in PH *Mogibacteriaceae*, *Ruminococcaceae* and *Prevotella* able to discriminate individuals with PD from PH
Dinakaran et al., 2019	Comparative study	To determine whether diseased full‐thickness colon specimens contain specific oral and gut pathogens	13 CD, 13 UC	NA	NA	Colonic mucosa specimens	16S rRNA sequencing (Illumina)	Proportion of non‐detrimental bacteria in CD or UC colon samples was altered compared to adjacent healthy colon specimens Microbiome of CD and UC diseased specimens is dominated by putative oral pathogens such as *Streptococcus*, *Staphylococcus*, *Peptostreptococcus*, *Fusobacterium*, *Prevotella, Porphyromonas, Veilonella and Eubacterium*
Strauss et al., 2011	Cross‐sectional	To determine whether *F. nucleatum* was associated with IBD patients and showed an increased capacity for invasion	17 CD, 4 UC, 1 IC, 34 HC (32 colon cancer screening, 2 IBS)	NA	NA	Colonic mucosa specimens	Fusobacterium‐selective agar plates (JVN plates): genomic DNA extracted from each isolate and used as template in a PCR	*Fusobacterium* spp. isolated from 63.6% of IBD patients versus 26.5% of HC The most recovered species was *F. nucleatum*, found in 50% of diseased patients and 17.6% of HC *F. nucleatum* from inflamed tissue of CD patients showed a significantly increased ability to invade Caco−2 cells in comparison with strains isolated from healthy tissue of both IBD and HC

Abbreviations: CD: Crohn's disease; CP: chronic periodontitis; CPITN: community periodontal index of treatment needs; G: gingivitis; GCF: gingival crevicular fluid; GI: gingival index; HC: healthy controls; IBD: inflammatory bowel diseases; IBS: irritable bowel syndrome; IC: indeterminate colitis; PBS: papilla bleeding score; PCR: polymerase chain reaction; PD: periodontitis; PH: periodontal health; PI: plaque index; PPD: probing pocket depth; SoP: suppuration; UC: ulcerative colitis; VPI: visible plaque index.

Lourenςo et al., (2018) analysed the gut microbiome in stool samples of 7 individuals with clinically healthy periodontium, 14 presenting gingivitis and 23 presenting chronic periodontitis. They found a tendency to lower alpha diversity in the gut microbiome of patients with chronic periodontitis when compared to HCs or gingivitis. Firmicutes, Proteobacteria, Verrucomicrobia and Euryarchaeota were increased, whereas Bacteroidetes were decreased in patients with periodontitis compared to periodontally healthy individuals with non‐statistically significant differences.

The study of Dinakaran et al., (2019) evaluated the presence of oral pathogens in 39 full‐thickness colon specimens obtained during elective colon surgery in CD and UC patients; while adjacent healthy colon specimens of these patients were analysed as control group. The results showed that microbiome of CD and UC diseased specimens was dominated by oral pathogens belonging to phyla Firmicutes and Fusobacteria, while adjacent healthy specimens showed an increased abundance of phyla Bacteroidetes and Actinobacteria. Strauss et al., (2011) isolated *Fusobacterium* spp. from 63.6% of colonic mucosa of patients with IBD (17 CD, 4 UC and 1 indeterminate colitis) and from 26.5% of colonic mucosa of healthy controls, and the most common species was *Fusobacterium nucleatum*. Interestingly, *F*. *nucleatum* from inflamed bowel of CD patients showed a significantly increased predisposition to cell invasion.

### 3.3 Evidence for a shared immunological pattern

Five of the included studies evaluated possible common immunological features between IBD and periodontitis as outlined in Table 3.

**TABLE 3:** General overview of studies investigating immunological alterations in IBD and PD patients

Author	Study design	Aims	Subjects	Periodontitis definition	Periodontal assessment	Tissue sampled	Laboratory analysis	Key findings
Figueredo et al., 2017	Cross‐sectional	To assess cytokine expression in gingival and intestinal tissue from patients with IBD and PD	21 IBD and PD (10 CD and 11 UC; 8 active IBD, 13 remission IBD)	At least 10 teeth with PPD ≥ 5 mm and CAL ≥ 4 mm in at least 4 sites, in different teeth	Full‐mouth protocol: PPD, CAL, BoP, PI	Gingival tissue (21) and intestinal tissue (21)	Multiplex assay	IL−4, IL−10 and IL−21 expression significantly increased in gingival tissue of patients with active IBD Inflammation score (mean value of IL−1 β, IL−6, IL−21 and sCD40L) significantly higher in gingival tissue of patients with IBD activity Significant correlation between gingival and intestinal inflammation scores Significantly higher IL−23 and IFN‐ γ levels and lower IL−31 and TNF‐ α levels in gingival tissues rather than in gut
Schmidt et al., 2018	Cross‐sectional	To investigate concentrations of aMMP−8 within a group of patients with IBD	30 CD, 29 UC, 59 HC	Moderate or severe PD according to AAP	Full‐mouth protocol: PPD, CAL, PBS	Subgingival plaque and GCF	ELISA for aMMP8	Higher aMMP−8 concentration in IBD patients as compared to HC Only in CD, increasing severity of periodontitis associated with an increase in aMMP−8 concentration
Menegat et al., 2016	Cross‐sectional	To evaluate the expression of cytokines in gingival tissue and intestinal mucosa of patients having both PD and IBD	28 IBD and CP (18 CD and 10 UC)	At least 8 teeth with probing depth (PD) ≥ 5 mm and CAL ≥ 4mm in at least 4 sites, in different teeth	Full‐mouth protocol: PPD, CAL, BoP, PI	Gingival tissue (24) and intestinal tissue (12)	Multiplex assay	No differences in cytokine levels between CD and UC Higher levels of IL−17A, IL−17F, IL−22, IL−25, IL−33, IL−10, and INF‐ γ in gingival tissues as compared to intestinal mucosa In gingival tissue, cytokines formed the clusters: IL−25/IL−10/IL−33, IL−22/ IL−23/IL−6 and IL−6/IL−25/IL−33/IL−10 In intestinal mucosa, the clusters were: IL−6/IL−21/IL−10, IL−17A/IL−6/IL−21/IL−10, IL‐I7F/IL−33/IL−25 and IL−23/IL−2/IL−17A/IL−6/IL−21/IL−10
Said et al., 2014	Cross‐sectional	To analyse the salivary microbiome alterations in IBD patients	21 CD, 14 UC, 24 HC	NA	NA	Saliva	Immunoassays for LL−37, IgA, salivary lysozyme, total protein count	No significant difference in the total protein concentration in saliva of the CD and UC patients as compared to HC. Lower lysozyme level in saliva of both the CD and UC groups as compared to HC. Higher levels of IgA and LL37 in both CD and UC groups as compared to HC In saliva of CD and UC groups, higher level of IL−1β as compared to HC. Higher levels of IL−6, IL−8 and MCP−1 in saliva of UC group as compared to HC. Elevated TNF‐a level in CD group. Higher levels of IgA and MCP−1 in UC when compared to CD
Figueredo et al., 2011	Cross‐sectional	To characterize the expression of cytokines in the GCF and serum from patients with both untreated PD and IBD, and patients with untreated PD	15 both CD and CP, 15 both UC and CP, 15 CP	At least 5 inflamed sites with PPD of ≥ 5 mm and CAL loss of ≥ 3 mm in different teeth	PPD, CAL, presence of plaque, BOP	Gingival crevicular fluid	ELISA for IL−18; Luminex assay for IL−1b, IL−4, IL−6, IL−10, IL−12p40, IL−12p70, TNF‐ α and IFN‐γ	Highest IL−4 from shallow sites of control group Lower IL−4 from deep sites of CD; higher IL−6 from deep sites of UC, as compared to control group Lower IL−4 from shallow sites of UC; lower IL−18 from shallow sites of CD, as compared to control group No significant difference between CD and UC IL−10, IL−12p40, IL−12p70 and TNF‐α below the levels of detection in gingival crevicular fluid In serum, lowest IL−18 in control group

Abbreviations: AAP: American Academy of Periodontology; BoP: bleeding on probing; CAL: clinical attachment loss; CD: Crohn's disease; CP: chronic periodontitis; G: gingivitis; GCF: gingival crevicular fluid; GI: gingival index; HC: healthy controls; IBD: inflammatory bowel diseases; PBS: papilla bleeding score; PCR: polymerase chain reaction; PD: periodontitis; PH: periodontal health; PI: plaque index; PPD: probing pocket depth; SoP: suppuration; UC: ulcerative colitis; VPI: visible plaque index.

Menegat et al., (2016) and Figueredo et al., (2011, 2017) compared gingival and intestinal cytokine expression in patients affected by both IBD and chronic periodontitis. Significantly higher levels of IL‐17A, IL‐17F, IL‐22, IL‐23, IL‐25, IL‐33, INF‐γ and IL‐10 in gingival tissue were detected, as well as a tendency for higher levels of IL‐6, IL‐31 and IL‐21. IL‐31 and TNF‐α were significantly increased in intestinal tissue. In gingival tissue, IL‐1β, IL‐4, IL‐10 and IL‐21 exhibited significantly higher levels in patients with active IBD. Overall, the cytokine clustering pattern was different in gingival and intestinal tissues. An inflammation score was calculated based on four cytokines, IL‐1β, IL‐6, IL‐21 and sCD40L, that were expressed in higher levels in active IBD. The score was significantly higher in intestinal tissue from patients with IBD activity and increased in gingival tissue from patients with IBD activity compared to patients in remission.

In a study by Schmidt et al., (2018), concentrations of aMMP‐8 were measured in gingival crevicular fluid (GCF) samples and the mean value was significantly higher in IBD compared to healthy controls; for CD patients, aMMP‐8 concentrations increased significantly with increasing severity of PD. Consistently, aMMP‐8 concentration in patients with no or mild PD and CD was comparable to healthy controls and was lower than in UC.

Said et al., (2014) analysed salivary IgA, cytokines, and enzymes comparatively in IBD patients and healthy controls. Lysozyme level was significantly lower in saliva of IBD patients when compared with healthy controls. On the other hand, the levels of IgA, LL37 (cathelicidin) and IL‐1β of IBD patients were higher than levels of the control group. The levels of IL‐6, IL‐8 and MCP‐1 were significantly higher in saliva of UC group only, while elevated TNF‐α level was found only in the CD group.

## 4 DISCUSSION

Recent systematic reviews have shown that patients with IBD are at a significantly higher risk of having periodontitis compared to non‐IBD patients, although the linking mechanisms need to be further explored (Papageorgiou et al., 2017; She et al., 2020). This paper aimed to evaluate the current level of evidence in human studies about biological mechanisms underlying the epidemiological association between the two diseases. Due to the high heterogeneity in objectives and methods across the selected studies, the findings are presented under the form of a scoping review. It was attempted to provide an overview of the state of art using a systematic search method, differing from systematic reviews in broadness of the coverage.

### 4.1 Oral microbial alterations in IBD patients

A distinct salivary and subgingival ecotype is detectable in patients with IBD patients compared to HCs. In the presence of UC and CD, physiologic oral microbiota is altered with characteristic features of dysbiotic states: bloom of pathobionts, depletion of commensals and overall loss of diversity (Korem et al., 2015). Remarkable changes in numerous oral microbial residents were found consistent with changes in the intestine of patients with IBD. Recent evidence has revealed a strong concordance between oral and gut microbiota in association with diseases such as liver cirrhosis and rheumatoid arthritis (Qin et al., 2014; Zhang et al., 2015). Furthermore, patients suffering from gut diseases display an aberrant enrichment of characteristic oral bacteria in both lumen and gut mucosa. Therefore, it can be hypothesized that oral cavity acts as a reservoir of pathobionts whose oral–gut translocation plays a role in the pathogenesis of IBD. On the other hand, periodontitis is likely to exacerbate gut inflammation and mediate inflammatory conditions at the systemic level (Kitamoto, Nagao‐Kitamoto, Hein, et al., 2020). Indeed, periodontitis and its chronic low‐grade inflammatory burden have been associated with several systemic conditions ultimately increasing the risk of mortality (Romandini et al., 2018, 2021).

Subgingival plaque from IBD patients revealed a higher prevalence of opportunistic bacteria, including *P. micra*, *T. denticola* in IBD patients and *Campylobacter* species (Wolinella in the old taxonomy). *Campylobacter* species are among the most frequent causes of bacterial gastroenteritis and were detected with a high prevalence both in intestinal biopsies and faecal samples of IBD patients compared to healthy subjects (Man, 2011).

Despite the evidence of taxonomic and functional dysbiosis, data on specific bacteria were controversial. A possible explanation may be found in the high inter‐individual variance when dealing with physiologic or pathologic microbiome, plausibly due to the differences in consumed food, age, and ethnic background (Dethlefsen et al., 2007). Furthermore, different detection techniques and different sequencing platform used in different studies might have generated discrepancies in the outcomes. Significant differences were also encountered between CD and UC patients, and even though CD and UC have important characteristics (such as symptoms, structural damage and therapy) in common, emerging evidence suggest that they represent two distinct pathophysiological entities (de Souza & Fiocchi, 2016).

Another subset of papers accounted for oral microbial alterations in paediatric patients with IBD. Although periodontitis was absent and no study presented a longitudinal follow‐up, the findings revealed oral dysbiotic alterations in children consistent with the ones found in adults (Kelsen et al., 2015).

### 4.2 Intestinal dysbiosis in periodontitis patients

Recent data have indicated that resident oral microbiota is able to colonize the gastrointestinal tract through haematogenous and enteral routes (Kitamoto, Nagao‐Kitamoto, Hein, et al., 2020). This intestinal translocation of bacteria from oral origin triggers inflammation and has been correlated with several non‐communicable diseases, including IBD, irritable bowel syndrome and colorectal cancer (Forbes et al., 2016). *P. gingivalis* is an important oral pathobiont, capable of cell invasion and resistant to gastric acidity (Hajishengallis et al., 2020). Oral administration of *P. gingivalis* to mice is reported to disrupt the gut epithelial integrity and to significantly alter microbial composition, both via outgrowing in the gut and systemic endotoxemia (Nakajima et al., 2015). Furthermore, *Klebsiella* spp., mainly isolated from the oral microbiota, are known to be strong inducers of T helper 1 (TH1) when they translocate to the gut (Atarashi et al., 2017).

Notably, genera as *Fusobacterium, Prevotella, Streptococcus* and *Porphyromonas* not only dominate the gut microbiome of patients with IBD but are also significantly more prevalent in diseased compared to adjacent healthy colon specimens (Dinakaran et al., 2019). Plausibly, these oral bacteria can act as keystone pathobionts when they colonize the gut native microbiota, with the effect of promoting a reduction in alpha diversity, which is considered as a reliable indicator of disease‐associated dysbiosis (Duvallet et al., 2017). In relation to beta diversity, it was not possible to clearly distinguish individuals with distinct clinical status based on the composition of the gut microbiota, as it resulted from the study of Lourenςo et al., (2018). However, this latter was a pilot investigation and presented a limited sample size. Not specific pathogens but rather a synergy of bacteria is implicated in IBD pathogenesis, and a unique dysbiotic microbiome cannot explain the aetiology of this disease (Duvallet et al., 2017). Notably, many consortia of bacteria are present in both healthy and diseased state. Also, gut mucosal microbial colonization may be significantly different from the one found in the lumen, with the former being more likely to interact and trigger the immune system (Li et al., 2015). Sampling from different parts of the intestine may provide more accurate reflection of the whole microbiome.

The relationship between intestinal dysbiosis and periodontitis may have a bidirectional character. The richness in the variety of gut bacteria can potentially affect the immune fitness of the entire body, by epigenetic remodelling and altered gene expression (Levy et al., 2017). The recent pandemic emergence of some multifactorial inflammatory, autoimmune, neurodegenerative and metabolic diseases has been consistently associated with overabundance of opportunistic pathogens in the gut (Ribaldone et al., 2019). Also, periodontitis is now investigated as an extraintestinal manifestation of enteric dysbiosis (She et al., 2020).

### 4.3 Evidence for a shared immunological pattern

IBD and periodontitis are both considered as a disproportionate mucosal inflammatory reaction to a microbial environment in susceptible patients, with host immune response playing a pivotal role (Agossa et al., 2017). Majority of the evidence comes from studies comparing the patterns of inflammatory cytokines expressed in gingival and intestinal tissues in patients affected by both periodontitis and IBD. Higher expressions of MMP‐8, IL‐17A and INF‐γ in the periodontal tissues of IBD patients encountered across the included studies may increase susceptibility to periodontal inflammation (Arias‐Bujanda et al., 2019). Similar findings were reported for patients suffering from rheumatoid arthritis and diabetes (Kirchner et al., 2017; Mohamed et al., 2015). Lack of consistency in the available data disables clarification of the true nature of the impact of IBD on periodontal health. Confounding factors such as smoking and IBD severity together with the medications affect the expression levels of cytokines and may be sources of biases. It is quite clear that IBD activity significantly influences gingival inflammatory status.

Most studies emphasized the role of Th17 cytokines, such as IL‐17A, IL‐21, IL‐22 and IL‐23 that sustain chronic periodontal lesions and are implicated in the aetiopathogenesis of IBD (Fujino et al., 2003; Gaffen et al., 2008). The similarity between IBD and periodontitis in terms of cytokine expression pattern may indicate an integrated mucosal immunological system and support the hypothesis that the two diseases share some common pathogenic mechanisms (Gill et al., 2010; Lira‐Junior & Figueredo, 2016). Accordingly, a recent preclinical study demonstrated generation of oral pathobiont‐reactive Th17 cells in the oral cavity by periodontitis and these cells may migrate to the gut via lymphatic routes. Once in the intestine, these cells can be activated by translocated oral pathobionts and trigger colitis (Kitamoto et al., 2020).

### 4.4 Limitations and future directions

The present work presents some inherent limitations, mainly ascribed to the available literature. The methodological heterogeneity of studies makes comparisons difficult. Most studies adopted an exploratory cross‐sectional design with poorly characterized samples in terms of both clinical periodontal assessment and IBD severity/activity.

Analysis of the effect of shared environmental factors was beyond the scope of the present review, as well as the role of background genetic susceptibility. Smoking, diet and Western lifestyle are prominent risk factors for IBD and that some genes associated with mucosal barrier function, antimicrobial recognition and function, or immune regulation can be involved, but evidence is scarce (for a review, see Ozmeric et al., 2018). On the other hand, emerging data suggest that role of fungi and viruses should not be neglected in both IBD and periodontitis pathogenesis, although the majority of studies referred to bacterial dysbiosis. Nevertheless, the causal pathway triggered by these genetic and environmental factors always points out to the dynamic interplay between the host immune component and the dysbiotic microbiome. The complexity of this relationship warrants further research. It remains unclear whether dysbiosis is a direct cause of the disease manifestation or whether the alterations in the microbial landscape are a consequence of alterations in the immune system, metabolism or diet (Levy et al., 2017). Future research endeavours should focus on both taxon‐ and function‐based microbiological analyses of oral and gut metagenome of IBD and periodontitis patients. Animal models dealing with microbiome transfer from gnotobiotic mice to germ‐free mice seem to be relevant to better clarify the issue. At the same time, the integration of diverse omics techniques, such as epigenomics, proteomics, transcriptomics or metabolomics, applied to saliva, GCF, plasma and stool could add important aetiopathologic and diagnostic information for establishing diverse biological phenotypes of both diseases (Baima et al., 2021). Longitudinally follow patients with IBD by a multiple biomarker approach could allow for both the monitoring of local/systemic inflammatory status and for identifying sites at risk for periodontal breakdown in this susceptible category of patients.

Efficient preventive and therapeutic measures against periodontal infection and building a closer collaboration between gastroenterologists and periodontal care providers may help to control intestinal inflammation in IBD and prevent severe gastrointestinal pathologies (Xun et al., 2018). Moreover, focusing on the microbiome in chronic inflammatory diseases may reveal new dimensions of their aetiology and may lead to the development of novel microbiome‐targeted therapeutic strategies (i.e. probiotics or faecal microbiome transplantation).

It is too early to conclude whether periodontitis and IBD are distinct disease entities interacting or manifestations of a general homoeostasis disruption between the immune system and the metagenome. Oral dysbiosis may precede gut dysbiosis, or both conditions may develop concomitantly on the basis of a common immune/inflammatory predisposition. Possible beneficial effects of periodontal treatment on gut dysbiosis or IBD clinical course deserve investigation (Agossa et al., 2017; Romano et al., 2019). At the same time, the impact of IBD drugs and probiotics therapies on the periodontal condition needs to be determined yet. Interventional trials with the concomitant analysis of clinical, microbiological and immunological markers of disease activity should be implemented to make significant advances in this field.

In conclusion, findings of the present review indicate that oral microbiome of patients with IBD is characterized by a lower diversity and higher dysbiotic traits compared to healthy subjects. Moreover, the data show that gut lumen and mucosal tissues of patients with IBD harbour periodontal pathogens that may have translocated via haematogenous and/or enteral route. There are animal studies showing that oral–gut translocation of pathobionts may cause intestinal dysbiosis but the clinical evidence is limited to observational studies. At present, a relationship in terms of an association seems to be likely and further epidemiologic cohort studies are warranted to determine whether a causal relationship exists between the two diseases. Considering the available immunological data, MMP‐8, IL‐17A, and INF‐γ seem to be promising biomarkers for an interaction between IBD and periodontitis and possible role of Th17 cells deserves further research.

## CONFLICT OF INTEREST

The authors declare that they have no competing interests.

## AUTHOR CONTRIBUTIONS

Giacomo Baima, Conceptualization; Data curation; Formal analysis; Investigation; Methodology; Project administration; Supervision; Validation; Writing‐original draft; Writing‐review & editing. Alessandro Massano: Data curation; Formal analysis; Investigation; Methodology; Resources; Validation; Writing‐original draft. Erminia Squillace: Formal analysis; Writing‐original draft. Gian Paolo Caviglia: Conceptualization; Supervision; Validation; Visualization. Nurcan Buduneli: Conceptualization; Validation; Visualization; Writing‐review & editing. Davide Giuseppe Ribaldone: Conceptualization; Methodology; Supervision; Writing‐review and editing. Mario Aimetti: Conceptualization; Project administration; Writing‐review and editing.

### PEER REVIEW

The peer review history for this article is available at https://publons.com/publon/10.1111/odi.13843.

## Supporting information

Fig S1

Table S1

## Data Availability

All data generated or analysed during this study are included in this published article (and its supplementary information files).
