# Supplementary figures and images for: Shared microbiological and immunological patterns in periodontitis and IBD: A scoping review
Source: Oral Dis. 2021 Mar 23;28(4):1029–41. doi: 10.1111/odi.13843 (PMC9291827; doi:10.1111/odi.13843)

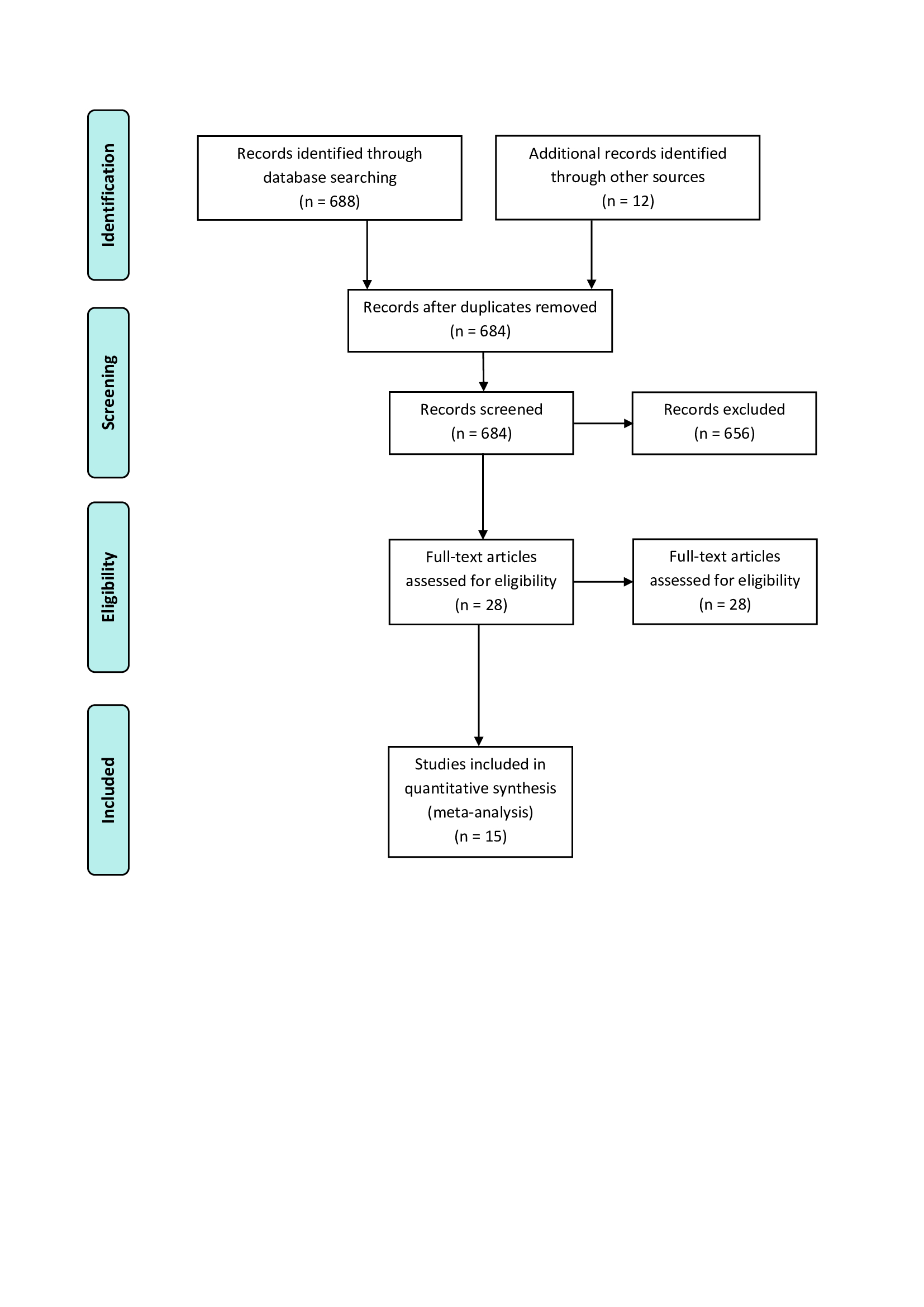

Supplement: Supplementary file 1 — Fig S1 [file ODI-28-1029-s001.jpg]
