# Supplementary material for: Shared microbiological and immunological patterns in periodontitis and IBD: A scoping review
Source: Oral Dis. 2021 Mar 23;28(4):1029–41. doi: 10.1111/odi.13843 (PMC9291827; doi:10.1111/odi.13843)
Supplement: Supplementary file 2 — Table S1 [file ODI-28-1029-s002.docx]

**Supplementary Table 1.** Studies excluded after full text reading, with reason for exclusion.

| **Author** | **Title** | **Reason for exclusion** |
| --- | --- | --- |
| Lamster et al.,  1978 ^1^ | An association between Crohn's disease, periodontal disease and enhanced neutrophil function | Case report |
| Engel et al.,  1988 ^2^ | Abnormal lymphocyte profiles and leukotriene B4 status in a patient with Crohn's disease and severe periodontitis | Case report |
| Flemmig et al.,  1991 ^3^ | Prevalence and severity of periodontal disease in patients with inflammatory bowel disease | No microbiological or immunological assessment |
| Grössner-Schreiber et al.,  2006 ^4^ | Prevalence of dental caries and periodontal disease in patients with inflammatory bowel disease: A case-control study | No microbiological or immunological assessment |
| Kaner et al.,  2006 ^5^ | Gingival crevicular fluid levels of calprotectin and myeloperoxidase during therapy for generalized aggressive periodontitis | No IBD or gastrointestinal bacteria |
| Brito et al.,  2008 ^6^ | Prevalence of periodontitis and DMFT index in patients with Crohn's disease and ulcerative colitis | No microbiological or immunological assessment |
| Loos et al.,  2009 ^7^ | NOD1 gene polymorphisms in relation to aggressive periodontitis | IBD |
| Kaner et al.,  2011 ^8^ | Calprotectin levels in gingival crevicular fluid predict disease activity in patients treated for generalized aggressive periodontitis | No IBD or gastrointestinal bacteria |
| Habashneh et al.,  2012 ^9^ | The association between inflammatory bowel disease and periodontitis among Jordanians: A case-control study | No microbiological or immunological assessment |
| Lundmark et al.,  2015 ^10^ | Transcriptome analysis reveals mucin 4 to be highly associated with periodontitis and identifies pleckstrin as a link to systemic diseases. | No microbiological or immunological assessment |
| Piras et al.,  2017 ^11^ | Prevalence of Apical Periodontitis in Patients with Inflammatory Bowel Diseases: A Retrospective Clinical Study | No microbiological or immunological assessment |
| Lin et al.,  2018 ^12^ | Increased risk of ulcerative colitis in patients with periodontal disease: A nationwide population-based cohort study | No microbiological or immunological assessment |
| Kato et al.,  2020 ^13^ | History of Inflammatory Bowel Disease and Self-Reported Oral Health: Women's Health Initiative Observational Study | No microbiological or immunological assessment |

***References***

1. Lamster I, Sonis S, Hannigan A, et al. An association between Crohn's disease, periodontal disease and enhanced neutrophil function. *J Periodontol* 1978 Sep; 49: 475-9.

2. Engel LD, Pasquinelli KL, Leone SA, et al. Abnormal lymphocyte profiles and leukotriene B4 status in a patient with Crohn's disease and severe periodontitis. *J Periodontol* 1988; 59: 841-7.

3. Flemmig TF, Shanahan F, Miyasaki KT. Prevalence and severity of periodontal disease in patients with inflammatory bowel disease. *J Clin Periodontol* 1991; 18: 690-7.

4. Grössner-Schreiber B, Fetter T, Hedderich J, et al. Prevalence of dental caries and periodontal disease in patients with inflammatory bowel disease: a case-control study. *J Clin Periodontol* 2006; 33: 478-84.

5. Kaner D, Bernimoulin JP, Kleber BM, et al. Gingival crevicular fluid levels of calprotectin and myeloperoxidase during therapy for generalized aggressive periodontitis. *J Periodontal Res* 2006; 41: 132-9.

6. Brito F, de Barros FC, Zaltman C, et al. Prevalence of periodontitis and DMFT index in patients with Crohn's disease and ulcerative colitis. *J Clin Periodontol* 2008; 35: 555-60.

7. Loos BG, Fiebig A, Nothnagel M, et al. NOD1 gene polymorphisms in relation to aggressive periodontitis. *Innate Immun* 2009; 15: 225-32.

8. Kaner D, Bernimoulin JP, Dietrich T, et al. Calprotectin levels in gingival crevicular fluid predict disease activity in patients treated for generalized aggressive periodontitis. *J Periodontal Res* 2011; 46: 417-26.

9. Habashneh RA, Khader YS, Alhumouz MK, et al. The association between inflammatory bowel disease and periodontitis among Jordanians: a case-control study. *J Periodontal Res* 2012; 47: 293-8.

10. Lundmark A, Davanian H, Båge T, et al. Transcriptome analysis reveals mucin 4 to be highly associated with periodontitis and identifies pleckstrin as a link to systemic diseases. *Sci Rep* 2015 21; 5: 18475.

11. Piras V, Usai P, Mezzena S, et al. Prevalence of Apical Periodontitis in Patients with Inflammatory Bowel Diseases: A Retrospective Clinical Study. *J Endod*; 43: 389-394.

12. Lin CY, Tseng KS, Liu JM, et al. Increased Risk of Ulcerative Colitis in Patients with Periodontal Disease: A Nationwide Population-Based Cohort Study. *Int J Environ Res Public Health* 2018 21; 15: 2602.

13. Kato I, Sun J, Larson J, et al. History of Inflammatory Bowel Disease and Self-Reported Oral Health: Women's Health Initiative Observational Study. *J Womens Health (Larchmt)* 2020; 29: 1032-1040.
